# Beyond behavioural change: prioritising structural solutions to control bacterial sexually transmitted infections

**DOI:** 10.1016/j.eclinm.2025.103198

**Published:** 2025-04-10

**Authors:** Jason J. Ong, Magnus Unemo, Jean-Michel Molina, Angelica E. Miranda, Kate L. Seib, Maeve Brito de Mello, Meg Doherty, Cheryl C. Johnson, Sinead Delany-Moretlwe, Christopher Fairley

**Affiliations:** a School of Translational Medicine, Monash University, Melbourne, Australia; b Melbourne Sexual Health Centre, Alfred Health, Melbourne, Australia; c WHO Collaborating Centre for Gonorrhoea and Other STIs, Örebro University, Sweden; d Institute for Global Health, University College London, London, UK; e University of Paris, France; f Federal University of Espirito Santo, Brazil; g Institute for Biomedicine and Glycomics, Griffith University, Australia; h WHO Global HIV, Hepatitis and STIs Programmes, Switzerland; i University of Witwatersrand, South Africa

**Keywords:** Sexually transmitted infections, Public health intervention, Diagnostics, Global health strategy

## Abstract

With over 374 million new curable bacterial STI cases annually, we are far from meeting global health targets. Despite their serious consequences for sexual, reproductive, and mental health, control efforts often focus on individual-level interventions like condom promotion and behavior change, which are insufficient. A scientific framework for STI control emphasizes reducing infectiousness, decreasing the number of susceptible individuals, and lowering transmission probability. Effective strategies should focus on environmental modifications, including expanding access to quality sexual health care, rapid testing with same-visit treatment, and AI-enhanced diagnostics. Equally critical are protecting susceptible communities through vaccination and chemoprophylaxis (e.g., doxycycline post-exposure prophylaxis). While individual behavioural interventions like condom promotion remain relevant, declining usage trends challenge their impact. Surveillance of STIs and antimicrobial resistance is essential, influencing all key drivers of transmission. To control STIs more effectively, we must shift from individual behaviour change to systems-level public health strategies. Prioritising accessible, stigma-free health services, leveraging technological advances, and investing in comprehensive public health policies will improve STI prevention and help meet global health goals.

**Funding:**

JJO, CKF and KS are funded by the Australian National Health and 10.13039/501100000265Medical Research Council (GNT1193955, GNT1172900 and GNT2017383, respectively).

## Introduction

The World Health Organization (WHO) estimated 374 million new cases of four curable bacterial sexually transmitted infections (STIs) in 2020: syphilis, gonorrhoea, chlamydia and trichomoniasis.^1^ Countries with strong STI surveillance systems have reported substantial increases in case notification rates in the past decade,^2^, ^3^, ^4^, ^5^, ^6^ further decreasing the likelihood of achieving the aspirational WHO global goal to end the epidemic of STIs by 2030: reducing the number of new infections of syphilis, gonorrhoea, chlamydia and trichomoniasis to less than 150 million per year.^7^

The control of STIs, particularly bacterial STIs has received little attention, despite their long-term health consequences.^8^ Inadequate burden of disease estimates mean that STI control is deprioritised in some settings because of competing priorities with diseases associated with higher mortality. In addition, the lack of government funding that is allocated to address STIs has been linked to them being perceived as self-inflicted and the result of poor personal choices. However, the largest cause of STI rates in the community is inadequate health services and not personal behaviours.^9^ Considering the geopolitical context with the politization of sexual health and ongoing stigma associated with STI, this scenario is unlikely to change.

Shifting the narrative of STI control away from poor personal choices that play relatively little role in their control towards improving access to health care may help gain some political buy-in. The lessons learned from COVID-19 epidemic has provided much greater level of understanding about the transmission of infectious disease in the community and government and may allow framing STIs within basic disease epidemiology concept.

## A framework to understand the science to control STIs

The basic reproductive number (R_0_) is a fundamental concept in epidemiology that describes the transmission potential of infectious diseases.^10^ R_0_ represents the average number of secondary infections produced by one infected individual in a fully susceptible population. R_0_ is used in mathematical models to understand the control and spread of STIs by predicting the impact of different control measures and can help guide resource allocation.^10^^,^^11^ Thus, to control STIs, the goal is to reduce R_0_ to below 1. R_0_ is influenced by three main factors.

1) Duration of infectiousness: the duration of time an individual with the STI remains infectious and can transmit the infection to others.
2) Contact rate: the rate and number of contacts an individual with the STI has with susceptible individuals.
3) Infectivity (transmissibility): the probability of transmission per contact between an individual with the STI and a susceptible individual.

**Fig. 1:**
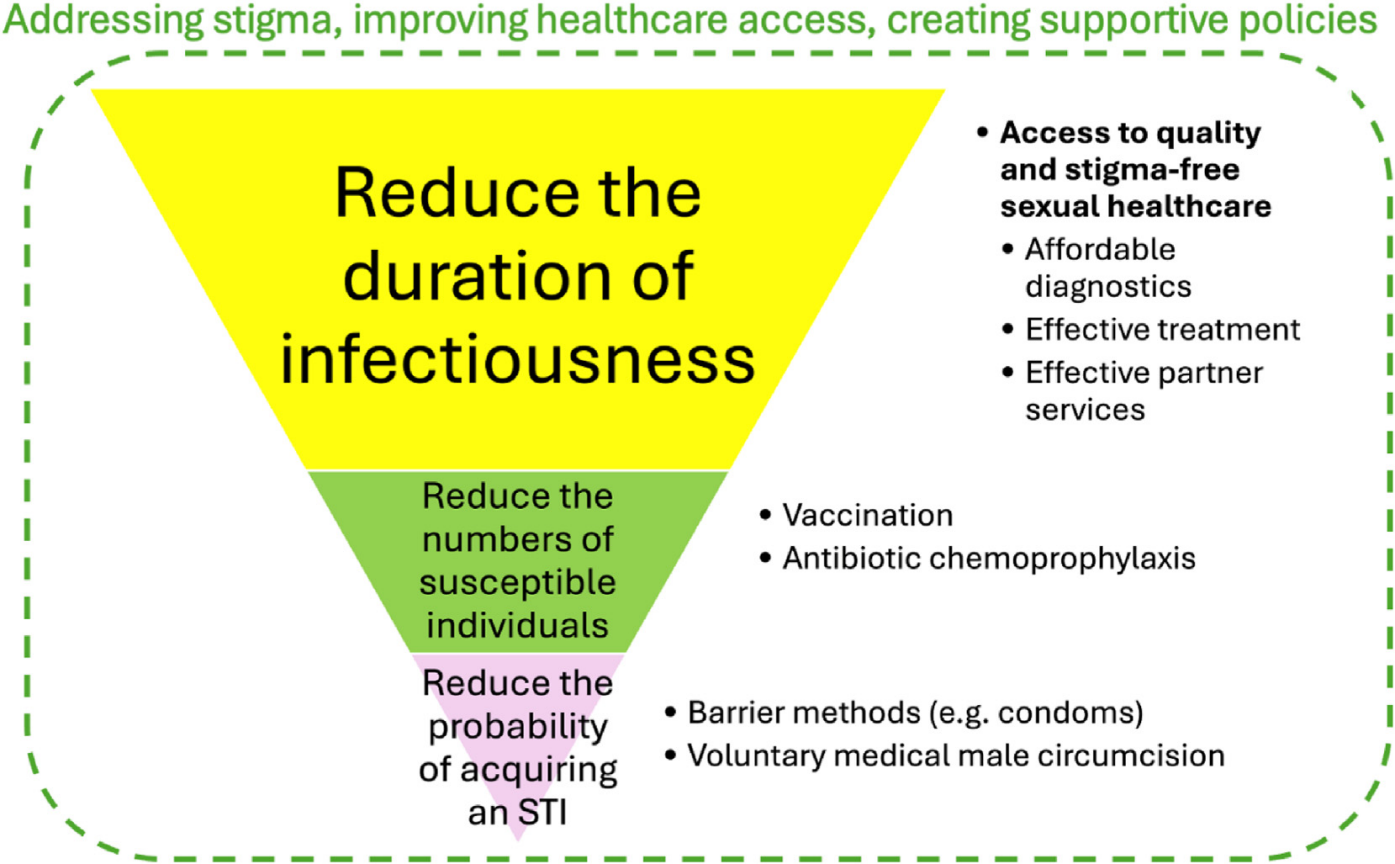
**Framework for controlling sexually transmitted infections^1^**.^1^ The proposed interventions can impact multiple drivers of STIs. For example, vaccination can also reduce the probability of acquiring an STI.

Fig. 1 illustrates how the framework can be applied to identify interventions for STI prevention and control. The focus is on implementing system-level changes that shape the environment in which individuals reside, rather than relying solely on changing individual behaviours. The following sections detail advancements in strategies to accelerate progress in STI control. While we categorise interventions under one of the three key drivers for clarity, we acknowledge that many interventions operate across multiple domains. For example, vaccines not only reduce susceptibility by providing immunity to vaccinated individuals but also lower the probability of acquisition indirectly through herd immunity by decreasing overall infection prevalence. Additionally, the effectiveness of these interventions is shaped by broader structural factors (stigma, barriers to healthcare access, policy constraints). Therefore, a comprehensive STI intervention should address stigma through community-led education campaigns and integrate STI services into general healthcare settings to enhance the uptake of interventions such as doxycycline post-exposure prophylaxis (doxyPEP) and vaccination.

While our framework aims to be broadly applicable across diverse populations, we acknowledge the significant role of core groups or key populations in STI transmission dynamics, highlighting the need for balanced resourcing between targeted and generalised interventions. When relevant, we will explicitly indicate where evidence is limited to specific core groups (e.g., doxyPEP among men who have sex with men (MSM)). Additionally, we emphasise that structural determinants—such as stigma, healthcare accessibility, and policy constraints—disproportionately affect key populations and shape intervention effectiveness.

## Reducing the duration of infectiousness

### Access to health care

When individuals at risk of STIs access health care, the testing and treatment they receive benefits them but also improves STI control at the community level. The higher their risk, the greater the benefit to STI control. Individuals with symptoms are also important because they are prompted to attend health care shortly after infection and therefore the treatment dramatically shorten the duration of infectiousness to only days.^9^ If these individuals cannot access treatment, and the symptoms resolve spontaneously, the duration of infectiousness can persist for long periods. It is no surprise that the introduction of penicillin in the UK in the 1940's saw a more than 2000% decline in syphilis cases.^9^ Scotland saw a similar significant decline in congenital syphilis over the same time. Even the decline in STIs associated with the appearance of the devasting HIV epidemic in the 1980s did not match the simple introduction of penicillin. Other countries also saw large declines in syphilis rates when penicillin was introduced.^9^

Therefore, syndromic management was recommended by the WHO for resource-constrained settings as a pragmatic approach for managing symptomatic STIs due to its feasibility in integration into primary health care, improving accessibility at the first point of contact and reducing stigma compared to visible STI-focused clinics.^12^ Although syndromic management plays an important role in symptom-based treatment, it does not replace broader STI control measures such as expanded testing, partner notification, and preventive interventions (e.g., vaccines, pre-exposure prophylaxis (PrEP), and doxyPEP).

Studies support the powerful effect of accessible health care and show lower rates of STIs when communities have greater access to health care. For example, indigenous Australians living in remote Indigenous communities had a 27-fold higher rate of trichomoniasis than individuals living in the closest city, despite both groups having a similar STI risk profile.^13^ A US study demonstrated a 39% lower STI risk for every four unit increase in the percentage of residents who had accessible primary health care.^14^

Access to healthcare is shaped by a complex interplay of structural, social, and economic factors that create significant disparities in STI prevention and treatment. Structural barriers, such as healthcare deserts—geographic areas with limited or no healthcare facilities—disproportionately affect marginalised communities, reducing access to timely diagnosis and treatment. Socioeconomic factors, including lack of insurance, high out-of-pocket costs, and transportation challenges, further limit healthcare utilisation, particularly for those in lower-income groups. Stigma and discrimination, especially against key populations such as LGBTQ + individuals, sex workers, and people living with HIV, discourage healthcare-seeking behaviour and reduce trust in medical institutions. Additionally, systemic issues like structural racism and gender inequities contribute to unequal treatment and access to services, further exacerbating health disparities. Digital and health literacy gaps also prevent individuals from effectively navigating healthcare systems and utilising digital health solutions, such as telemedicine or AI-assisted diagnostics. Addressing these barriers will require a multifaceted approach, including policy reforms, expansion of community-based services, stigma reduction initiatives, and equitable access to innovative healthcare solutions.

### Role of point-of-care testing

Accessible healthcare has been supported by bringing rapid STI testing closer to individuals to significantly reduce the duration of infectiousness compared with standard practices, which generally rely on syndromic management. Point-of-care tests (POCTs) or near-patient tests for STIs like HIV, syphilis, gonorrhoea, and chlamydia are a major public health advancement and their use has been growing globally.^15^, ^16^, ^17^ These tests can detect specific antigens or antibodies through assays and offer high sensitivity and specificity with molecular amplification methods. However, they present both significant opportunities and challenges in their implementation.^18^, ^19^, ^20^, ^21^

One of the key advantages of POCTs is their ability to deliver same-visit results and enable immediate treatment, especially in remote areas.^22^, ^23^, ^24^ This allows clients to swiftly access prevention and treatment services which are key to preventing the spread of infections.^19^ A study from Australia that used same-day near-patient POCTs for *Neisseria gonorrhoeae, Chlamydia trachomatis, and Mycoplasma genitalium* led to higher levels of appropriate treatment (80.9% vs. 33.0%) compared to routine testing, and 95.2% of individuals in the POCT group notified their sexual partners within 24 h, demonstrating how timely diagnostic results may facilitate rapid partner notification and potentially reduce further transmission.^25^ Similarly, a modelling study in Canadian arctic communities showed that POCTs can shorten the infectious period and reduce transmission by offering same-day testing and treatment.^26^

POCTs can be used in several settings, including community clinics, remote areas, mobile units, and even at home and as self-tests, improving access to testing in underserved areas. Bringing testing closer to those in need can decrease the gap in healthcare disparities and empower individuals by offering more privacy, reducing stigma associated with STI testing and encouraging more people to get tested, especially those who might otherwise avoid it due to fear of judgment or confidentiality concerns.^20^

While POCTs offer rapid results, they also face several challenges. As with any tests, accuracy can vary, with concerns about false positives and negatives, particularly when used in low-prevalence settings or populations. Ensuring tests meet quality standards for sensitivity and specificity is an important task. Integrating these tests into existing healthcare systems poses logistical challenges, such as recording results in medical records, linking them to appropriate care, and including them in public health data. Adequate training and quality control are essential for reliable results.^21^ Although POCTs can be cost-effective in the long-term, initial start-up costs, especially for some STIs, may be high and unaffordable in low-resource areas.^27^ Thus, ensuring equitable access, particularly in rural or vulnerable communities, remains a challenge.

### Role of partner notification

Active follow-up to provide testing and treatment to the partners of people with STIs is one of the most effective strategies for identifying new STI cases and breaking the chain of transmission.^28^^,^^29^ Such strategies are key for routine programming and outbreak responses. Most recently, partner services have been deployed as part of the Mpox response.^30^ When geographic outbreaks of syphilis^31^ and HIV^32^ emerged, such services have also been shown to slow and stop transmission by successfully identifying and treating cases early.

In the last decade, the scale-up of prompt provider-assisted referral in the context of HIV has had a significant impact globally,^33^ and lessons learned can be applied across bacterial STIs.^34^^,^^35^ Recent WHO guidance has also highlighted the role of broadening social network-based testing services by focusing on social and sexual networks.^36^^,^^37^ These services can reach people who are less likely to engage with the health system and may be more acceptable to those concerned about potential stigma or discrimination. Expedited partner therapy for curable STI is recommended in some settings and for some STIs but the effects and benefits may vary^29^^,^^38^^,^^39^; further, the approach can be costly and complex to implement,^40^^,^^41^ particularly in contexts where providers cannot prescribe for partners and where there are growing concerns about antimicrobial resistance (AMR).

A key challenge to implementing partner services for STIs is considering prioritising limited staff and financial resources. Integration of services is likely a high-impact and affordable option to consider, such as adding in additional services for STIs where a standalone service for HIV may otherwise be offered. High-impact settings and populations may also be an important way to focus—such as through targeting pregnant women and their partners, key populations and services where STI testing is being introduced such as PrEP and PEP programmes.^34^ The advent of self-testing and virtual interventions may also make this more feasible, particularly emerging multiplex products like the HIV/syphilis dual self-tests that can be considered.^42^, ^43^, ^44^

### Future improvements for self-assessment or risk and symptoms—the role of AI

If individuals with STI-related symptoms or potential exposure can test and receive treatment promptly, the duration of infectiousness can be reduced. To facilitate timely care-seeking, symptom checkers^45^^,^^46^ and online risk assessment tools^47^ are available to prompt individuals to seek healthcare services. Similarly, the rapid adoption of digital health technologies, including mobile health (mHealth) apps, web-based platforms, and telehealth services, is expanding access to sexual health care.^48^, ^49^, ^50^ However, these tools have not been formally evaluated to determine their impact on reducing duration of infectiousness.

With the recent emergence of artificial intelligence (AI) impacting many aspects of healthcare, the use of AI-assisted sexual healthcare is rapidly evolving. Specifically, there is great potential for improving self-care by expanding access to sexual health information, prompting health care seeking and earlier access to treatment.

AI-assisted self-assessment apps could reduce the duration of infectiousness by allowing individuals to accurately assess their risk for HIV/STIs and directing them to relevant services. For example, researchers from Melbourne Sexual Health Centre (MSHC), the largest public sexual health clinic in Australia, developed an AI-assisted self-assessment app that detects common STIs and other related anogenital skin conditions with reasonable accuracy.^51^^,^^52^ This web-based app may encourage individuals with STIs to present earlier than they might otherwise for examination and treatment. However, there is potential for misuse of AI-tools if its framing and limitations are not accurately and explicitly provided to the end user.^53^

There are no randomized controlled trials to evaluate the effectiveness or cost-effectiveness of incorporating emerging AI-tools in sexual health. There is also a paucity of evidence of patient preferences for how they would use AI-tools to optimize their sexual health. For instance, it is not clear how much trust they would place or whether clinician oversight and validation of AI results is still needed. Discrete choice experiments from Australia and the US found a preference for combined diagnosis from both AI and a clinician.^54^^,^^55^ However, as AI could outperform human diagnosis soon, this perception may change. Similarly, a discrete choice experiment of preferences for the use of an AI-based risk assessment tool found cost and accuracy were the drivers of the use of the AI-tool.^56^ If these tools are scaled up, we must ensure equal access to these AI-tools, such as accessing networks, cost of devices or data, having adequate skills and literacies. If these tools are to be scaled up, it is essential to ensure equitable access for everyone. This includes addressing barriers such as network availability, the affordability of devices and data, the need for adequate skills and digital literacy, and the need to invest in necessary infrastructure to support development and scale-up.

While AI offers promising solutions that could improve earlier testing and treatment of STIs, there is an urgent need for ensuring digital inclusion, strengthening its evidence base, developing clear governance and effective regulation to ensure its safety in the context of healthcare and clinical decision-making.

### How antimicrobial resistance could impact duration of infectiousness

During the recent century, antimicrobials have enabled effective treatment of STIs and, through reduction of the duration of infectiousness and potentially transmissibility, gave hope of control of the transmission of the STIs.^57^ However, emergence of AMR has challenged the effective management and control of STIs by increasing its duration of infectiousness by allowing antimicrobial-resistant bacteria to persist within a host for a longer period. Resistance in *N. gonorrhoeae* to the last remaining empiric first-line gonorrhoea treatment, ceftriaxone, is increasing^57^, ^58^, ^59^ and, due to AMR, incurable *M. genitalium* infections are already observed.^57^^,^^60^ Fortunately, *Treponema pallidum* has remained susceptible to the first-line syphilis treatment, benzathine penicillin G, and *C. trachomatis* resistance to first-line doxycyline and azithromycin has not be reported.^61^^,^^62^ However, searching for alternative as well as novel therapies remain important due to single supplier, risk of global stock outs of benzathine penicillin G, and AMR in gonorrhoea and *M. genitalium* infections.

Many complex factors have contributed to high levels of AMR in *N. gonorrhoeae* and *M. genitalium*. These include bacterial factors such as natural competence for transformation (*N. gonorrhoeae*) and a high mutational rate (both *N. gonorrhoeae* and *M. genitalium*). The AMR emergence in these STIs is then substantially potentiated by the extensive overuse and misuse of antimicrobials in general (causing a reservoir of AMR determinants in other bacterial species that can be acquired by the STI pathogens) as well as in STI treatment. This is sustained by misuse and overuse of antimicrobials, and use of antimicrobials of suboptimal quality and/or in suboptimal doses. There is growing concern about the routine screening and subsequent overuse of antimicrobials in certain populations (e.g., chlamydia and gonorrhoea screening for MSM)^63^ particularly when infections are asymptomatic and have limited clinical consequences. Moreover, evidence suggests that increasing screening coverage for chlamydia among young people may not effectively reduce population-level prevalence.^64^^,^^65^ In the absence of appropriate national and international surveillance of AMR in vitro, antimicrobial use (general and STI specific) and quality control, pharmacodynamics of antimicrobials, clinical efficacy of antimicrobials or treatment failures that support selection of optimal antimicrobial treatment regimen, the AMR situation can rapidly get out of control. Thus, use of resistance-guided individualised therapy (e.g., for *M. genitalium*^66^^,^^67^ and *N. gonorrhoeae**^68^*) or combination therapies (e.g., for *N. gonorrhoeae**^69^*) are aimed at preserving the efficacy of existing antibiotics and delaying the emergence of AMR. The current reliance on single treatments, such as with the ceftriaxone treatment of gonorrhoea, risks to select resistance to this last treatment option for gonorrhoea. Promisingly, the novel antimicrobials zoliflodacin^70^ and gepotidacin^71^ have recently shown non-inferiority compared to the recommended ceftriaxone-azithromycin dual therapy in phase 3 randomised controlled clinical trials. If these antimicrobials become licensed for the treatment of gonorrhoea, they will also be used and this will limit the selective pressure for ceftriaxone resistance. Finally, this situation is further fuelled by the high global prevalence and incidence of these STIs and limited effective disease-control measures in many global settings.^72^, ^73^, ^74^

## Reducing contact rate

### Chemoprophylaxis to protect people from an STI

The use of doxyPEP of bacterial STIs (chlamydia, syphilis, gonorrhoea) was first assessed in a randomized controlled trial (Ipergay) among MSM and transgender women (TGW) using oral PrEP for HIV prevention in France.^75^ Participants in the DoxyPEP arm were instructed to take a single oral dose of 200 mg of doxycycline ideally within 24 h after condomless sex (and no more than 72 h). This trial demonstrated a significant reduction of the occurrence of a first episode of chlamydia (hazard ratio (HR) 0.30) and syphilis (HR 0.27) but not of gonorrhoea (HR 0.83) in the DoxyPEP arm. These results were confirmed in two additional randomized trials using a similar design and conducted in the USA and France among MSM and TGW using HIV PrEP or living with HIV.^76^^,^^77^ In those trials, a significant reduction of STIs incidence was reported in the DoxyPEP arms with relative risks or hazard ratios vs. standard of care arms ranging from 0.12 to 0.26 for chlamydia, 0.13 to 0.23 for syphilis and, although to a lesser degree, from 0.43 to 0.67 for gonorrhoea (probably explained by the already high prevalence of doxycycline resistance in *N. gonorrhoeae*). Adherence to DoxyPEP was high with detection of doxycycline in urine (consistent with drug intake within a week) in 69% of participants at 6 months.^77^

Another trial with a similar design was conducted among young women using oral HIV PrEP in Kenya but did not show any significant impact of DoxyPEP on incidence of STIs.^78^ However, there was only a single case of syphilis in these women, all strains of *N. gonorrhoeae* in both arms were highly resistant to doxycycline, and importantly adherence to doxyPEP was low, with only 29% of visits among women randomized to the DoxyPEP arm with doxycycline detected in hair samples (consistent with drug intake in the previous month). So the results of this trial cannot dismiss the efficacy of DoxyPEP in women, which is plausible according to a pharmacokinetics study demonstrating high (above bacterial MICs) and prolonged (2–4 days) concentrations of doxycycline in blood, urine, vaginal and cervical swabs and biopsies in women following a single dose of 200 mg of doxycycline.^79^ In addition, a small study conducted among female sex workers in Japan reported an overall reduction of STIs with daily doxycycline prophylaxis suggesting that DoxyPEP might also be effective in this community.^80^ More data is needed in cis-women given the substantial burden of bacterial STIs by individuals of reproductive potential and the consequences on pregnancy and infant outcomes. A trial in the US (ATN 173) will evaluate post-exposure and weekly dosing among adolescents and young people assigned female at birth.

Following the results of these trials, a number of countries and guidelines (US CDC, European EACS, IAS-USA, Australasian) have recommended the use of DoxyPEP for MSM and TGW with a history of STIs, and in cities like San Francisco where the Department of Public Health was the first to issue guidance on the use of DoxyPEP, a strong decline (50%) in chlamydia and syphilis incidence but not gonorrhoea, was reported within a year following implementation.^81^, ^82^, ^83^, ^84^

One of the major issues with DoxyPEP is its impact on AMR. We already know that DoxyPEP can select for high level tetracycline resistance in *N. gonorrhoeae* (and the resistance to tetracycline is already high globally),^85^ but the real threat would be the selection of resistance to the third generation cephalosporin ceftriaxone. There is also a theoretical risk of selecting doxycycline resistance in chlamydia and syphilis (and *M. genitalium*) although there was no evidence so far in clinical trials, but follow-up was limited to one year on average. Longer-term follow-up is needed as well as careful monitoring of AMR in STI agents and other bacteria even though the impact on the gut microbiome seems limited.^86^ Adherence is a critical factor for DoxyPEP effectiveness. To address this, in-depth social science studies are needed to identify strategies for improving adherence. This could include exploring alternative delivery methods, such as weekly oral dosing or the development of topical formulations.

### Vaccinations

Vaccines are a key public health tool for long-term, sustainable control of infectious diseases, however effective vaccines are currently only available for the viral STIs such as hepatitis B virus (HBV) and human papillomavirus (HPV).^87^ The WHO recently identified vaccines as a top priority for STI control, particularly for *N. gonorrhoeae*, *Herpes simplex virus* (HSV), *T. pallidum* and *C. trachomatis*.^88^ STI vaccine candidates for these and other STIs are in various stages of the development pipeline, from early exploratory and antigen discovery stages (e.g., *M. genitalium*, trichomoniasis), to preclinical (e.g., syphilis^89^) and clinical development (e.g., gonorrhoea,^90^^,^^91^ chlamydia,^92^ HSV^93^).

Gonorrhoea vaccines have been prioritised for development because of the additional threat of AMR and the potential to mitigate the emergence of resistance to current antibiotics through antibiotic sparing. There is increasing interest regarding modest protection against *N. gonorrhoeae* that may be provided by the vaccine 4CMenB, which is licensed and widely used to protect against invasive meningococcal disease caused by the closely related bacteria *Neisseria meningitidis.* 4CMenB-induced antibodies from vaccinated individuals cross-react with *N. gonorrhoeae*,^94^ and several retrospective and prospective observational studies have estimated that 4CMenB provides 33–47% protection against gonorrhoea (reviewed in^95^^,^^96^).

Studies have seen reductions in gonorrhoea in various settings following the use of 4CMenB: 33.2% vaccine effectiveness against *N. gonorrhoeae* in adolescents and young adults in South Australia following implementation of an extensive 4CMenB programme^97^^,^^98^; 47% (13–68%) effectiveness in 18–29 year-old University students in Oregon, USA following mass vaccination campaigns prompted by group B meningococcal outbreaks^99^; 40% (25–53%) effectiveness in 16–23 year olds in New York City and Philadelphia, USA^100^; 44% (9–65%) effectiveness in MSM living with HIV in Milan, Italy^101^; and 46% (HR 0.54; 0.34–0.86) rate reduction in 15–30 year olds in California, USA.^102^ Modelling studies have also predicted that the use of a vaccine with modest efficacy, like 4CMenB, would have substantial impact on gonorrhoea prevalence (reviewed in^90^^,^^96^^,^^103^) and that the use of 4CMenB to prevent gonorrhoea is likely to be cost-effective, especially if used in communities at higher risk of infection.^104^^,^^105^ On the basis of evidence regarding potential impact and cost-effectiveness, the UK Joint Scientific Committee on Vaccination and Immunisation (JCVI) issued advice in November 2023 that a targeted programme should be introduced using 4CMenB to prevent gonorrhoea among individuals attending sexual health services in the UK who are at increased risk of infection, including gay, bisexual and other MSM (GBMSM).^106^ A dual indication targeting both gonorrhoea and meningococcal disease could help reduce stigma and promote adoption in countries with high prevalence of these conditions.

Unlike the observational studies, the recently completed DoxyVac randomised controlled trial in MSM aged 18 years or older in France did not see a significant reduction of gonorrhoea incidence following 4CMenB vaccination. There was a reported incidence of a first episode of gonorrhoea of 58.3 and 77.1 per 100 person-years in the 4CmenB vaccine group and no vaccine groups, respectively (aHR 0.78).^77^ There are several randomised controlled trials ongoing (reviewed in^96^) that will hopefully confirm whether 4CMenB is able to provide cross-protection against *N. gonorrhoeae* and address questions that remain regarding efficacy in different target groups and at different anatomical sites, as well as its potential duration of protection.

Even though WHO global stakeholder surveys identified vaccines as a priority for STI control, there was a large gap between perceptions of their public health relevance and their research feasibility.^88^ This is likely due to the many challenges that exist, including scientific knowledge gaps, the time and cost to develop a vaccine, complete clinical trials and implement vaccine programs.^87^^,^^107^ Public willingness to receive STI vaccines is also a consideration.^108^

When considering the role of vaccines in the framework for controlling STI, it is important to note that they can both directly and indirectly reduce the number of susceptible individuals. Vaccines provide direct protection by stimulating the body's immune response to protect against infection in vaccinated individuals. However, when vaccine uptake in the community is high enough, they can also provide indirect protection in unvaccinated individuals (i.e., herd immunity), by reducing the prevalence of infection. Indeed, HPV vaccination has had a substantial impact on HPV prevalence, genital warts and cervical precancer/cancer in both vaccinated and unvaccinated individuals.^109^ Therefore, in order to see the development and effective use of vaccines, consideration needs to be given scientific, logistic, economic, and social issues associated with vaccination.

## Reducing infectivity (transmission)

### Condoms

Male condoms (and to a lesser extent female condoms) are relatively effective and cheap barrier methods to reduce the probability of acquiring an STI. With the beginnings of the AIDS pandemic, there was a rapid rise in consistent use of condoms. However, in recent years, consistent condom use has fallen substantially in many countries. Reasons for this are complex and may be partly explained by the removal of fear of HIV due to treatment as prevention and PrEP for HIV, reduced investments in condom campaigns, and changes in social norms around sexual behaviours.^9^ Where PrEP is being scaled up, multiple studies have observed a higher incidence of bacterial STIs among individuals using PrEP which may be driven by more regular STI screening and/or less consistent condom use.^110^ In some countries, supply and cost challenges persist due to the lack of local condom manufacturing infrastructure.

According to the UNAIDS Global AIDS Monitoring report, in 2023, 56% of men aged 15–49 years reported condom use with non-regular partner (target is 80%). For MSM, the number was slightly higher (63%) that reported condom use at last anal sex with another man. However, this varied significantly by country (from 26% in Tanzania to 95% in India). Overall, the global trends are declining over time.^111^

The female condom (FC) only accounts for 1.6% of total condom distribution worldwide, despite evidence from laboratory studies demonstrating that it is as effective as the male condom in protecting against HIV and other STIs.^112^^,^^113^ Clinical studies have also shown that female condoms significantly reduce the risk of HIV and other STIs when used consistently and correctly. Partner acceptability, functionality, aesthetics, and access have been identified as key barriers to female condom uptake.^113^

Although there is scope to improve consistent condom use, its effectiveness relies on consistent use at every sex act, which may be challenging to negotiate in situations where there is a power imbalance. People do not consistently use condoms due to a mix of psychological, social, and situational factors. Cognitive dissonance, present bias, and optimism bias lead to underestimating risks and prioritizing immediate pleasure. Alcohol and drugs impair judgment, while peer pressure and social norms discourage use. Emotional factors like fear of rejection and ambivalence also play a role. Habitual behaviours, inadequate planning, and overreliance on alternative protection also contribute.

Improving condom use involves comprehensive sexual education, increased accessibility and enhancing condom design. Behavioural interventions, social marketing, and open partner communication are crucial. Addressing cultural barriers, empowering individuals, and targeting high-risk groups can support consistent condom use. A randomised controlled trial of personalised risk-reduction plans can increase condom use and prevent new STIs.^114^ However, whilst it is important to offer people the choice to use condoms, their incremental benefit compared to other strategies discussed might be marginal. Further complicating this issue is the current precipitous decline in condom use that has occurred with the introduction of PrEP for HIV, indicating that increasing condom use in the current environment will be challenging.

### Voluntary medical male circumcision

Male circumcision may reduce the risk of acquiring some viral STIs, particularly HIV, HSV-2 and HPV. A large randomised controlled trial in South Africa demonstrated that circumcised men had a 60% lower risk of acquiring HIV compared to uncircumcised men,^115^ leading the WHO and UNAIDS to recommend voluntary medical male circumcision (VMMC) as part of comprehensive HIV prevention strategies since 2007.^116^ Circumcision reduces the risk of HSV-2 infection by 28% and decreases the likelihood of high-risk HPV genotypes by 35%.^117^ The protective effect is attributed to removing the foreskin, which harbours a high density of Langerhans cells—target cells for viral entry—and creates a moist environment conducive to pathogen survival. In addition, female partners of circumcised men experienced lower rates of trichomoniasis and bacterial vaginosis.^118^ While male circumcision does not provide complete protection, it might be an effective intervention that, when combined with condom use, STI testing, and other biomedical strategies, contributes to broader STI prevention efforts. However, its implementation should be guided by cultural, ethical, and individual choice considerations to ensure uptake in different populations.

## A practical approach to the control of STIs

To effectively control STIs and contain AMR, urgent action is needed at the intersection of public health policy, research, and clinical practice. While scientific advancements provide a robust foundation for understanding and managing bacterial STIs, addressing the growing global burden requires a holistic, multi-sectoral approach as the implementation of effective strategies is often hindered by inadequate resources and competing public health priorities. Advocacy and public engagement are crucial in driving governmental funding decisions. Societal norms and stigma surrounding sexual and reproductive health can suppress advocacy efforts, reducing the political will to prioritise STI funding. Thus, governments and healthcare systems must prioritise accessible, quality, and stigma-free sexual health services (gender-inclusive and non-discriminatory), ensuring the availability and sustainability of comprehensive testing, treatment, and partner notification strategies. STIs are not evenly distributed within communities so STI control programs need to find ways to reach those least engaged in care and potentially most marginalised and stigmatised. Investment in infrastructure, particularly in underserved regions, is critical to close gaps in care, reduce infection rates, and address the rising threat of AMR. Denmark serves as an example of how comprehensive sexual health education, a robust primary healthcare infrastructure, and sustained public investment in sexual health services can result in relatively better STI control compared to many other countries.^119^

**Fig. 2:**
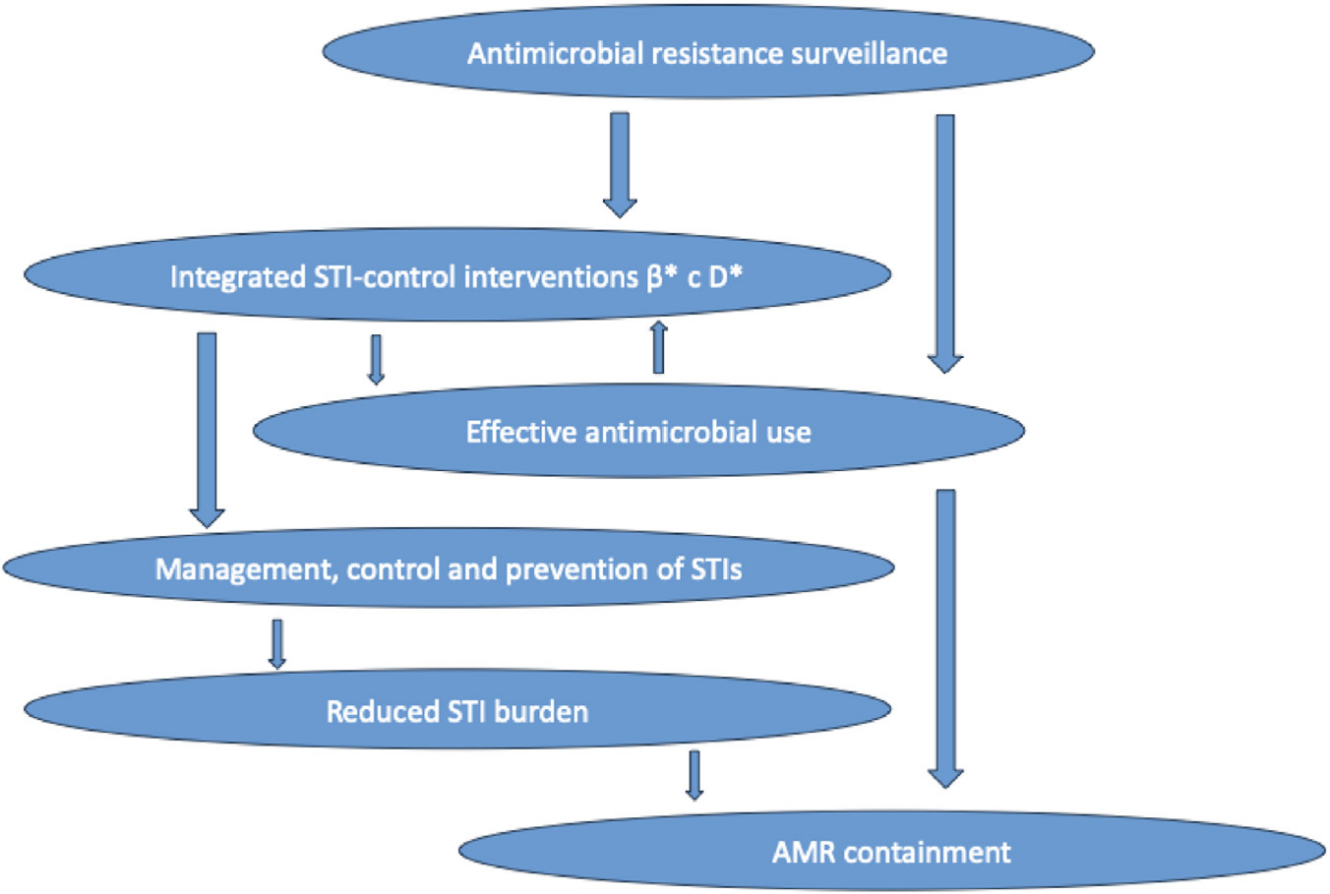
The relationships between antimicrobial resistance (AMR) surveillance for the public health purposes of optimizing treatments of sexually transmitted infections (STIs) and for establishing interventions for control of STIs as well as AMR.^72^ β, transmissibility; c, rate of partner exchange; D, duration of infectiousness; ∗, factors affected by effective antimicrobial treatment.

The relationships between AMR surveillance for the public health purposes of optimizing STI treatments and interventions for control of STIs and AMR are summarized in Fig. 2. Briefly, appropriate and quality-assured AMR surveillance is imperative to timely optimize evidence-based treatment guidelines, which should result in effective and timely antimicrobial use (availability and affordability of high-quality antimicrobials in all settings are essential), as well as to improve integrated STI-control interventions that enhance STI prevention, management, and control to reduce the burden of these STIs. Finally, both the effective antimicrobial use and reduced burden of these STIs are important for an effective AMR containment. For gonorrhoea, WHO attempts to support this through the global *N. gonorrhoeae* AMR surveillance (i.e., WHO GASP^58^/EGASP^120^, ^121^, ^122^, ^123^, ^124^/GLASS^58^), AMR surveillance for additional priority pathogens (GLASS), and optimized management guideline for gonorrhoea and other STIs.^125^

## Conclusions and outstanding questions

Interventions to control STIs will be most effective if they focus broadly on accessible health care rather than individual behavioural interventions, and this is particularly true if the interventions reduce the pleasure associated with sex. Health care should include not just clinics that provide free-of-charge or affordable testing and recommended and affordable treatment but reduce stigma and include improved health literacy including web-based self-diagnosis, point of care testing, vaccinations, partner treatment interventions, and biomedical interventions such as doxyPEP and others discussed in this review.

Following the science and rapidly translating technological and methodological innovations into practical solutions for STI prevention, diagnosis, and management will be pivotal in achieving WHO global targets.^7^ Generating global visibility around the public health challenges posed by STIs (including costs to health systems and their sequelae) and fostering political will through highlighting the financial and social benefits of investing in STI control strategies are critical next steps. By adopting this comprehensive approach, we can work towards optimising treatment outcomes and reducing the global burden of STIs.

## Contributors

JO conceived the idea for the review and led the writing. All authors contributed to the development of the review framework and critically revised multiple drafts of the manuscript. All authors approved the final version of the manuscript.

## Declaration of interests

MM, MD and CJ are staff members of the World Health Organization. The authors alone are responsible for the views expressed in this publication, and they do not necessarily represent the decisions, policy, or views of the World Health Organization. JM has received grants from Merck and Gilead (payment to his institution) and personal payments from Gilead, Merck and Viiv for consulting fees. MU has received research grant from the Global Antibiotic Research & Development Partnership (GARDP). JO has received personal payments from Gilead to present plenary talks at the Hong Kong Society of HIV Medicine Scientific Meeting (2024) and Taiwan AIDS Society Conference (2024).
